# Matrix metalloproteinase 7 promoted Schwann cell migration and myelination after rat sciatic nerve injury

**DOI:** 10.1186/s13041-019-0516-6

**Published:** 2019-12-02

**Authors:** Hongkui Wang, Ping Zhang, Jun Yu, Fuchao Zhang, Wenzhao Dai, Sheng Yi

**Affiliations:** 1 Key laboratory of neuroregeneration of Jiangsu and Ministry of Education, Co-innovation Center of Neuroregeneration, Nantong University, Nantong, Jiangsu China; 2 Faculty of Brain Science, University College London, Gower Street, London, WC1E 6BT UK

**Keywords:** Peripheral nerve injury, MMP7, Schwann cell, Migration, Myelination

## Abstract

Schwann cells experience de-differentiation, proliferation, migration, re-differentiation and myelination, and participate in the repair and regeneration of injured peripheral nerves. Our previous sequencing analysis suggested that the gene expression level of matrix metalloproteinase 7 (MMP7), a Schwann cell-secreted proteolytic enzyme, was robustly elevated in rat sciatic nerve segments after nerve injury. However, the biological roles of MMP7 are poorly understood. Here, we exposed primary cultured Schwann cells with MMP7 recombinant protein and transfected siRNA against MMP7 into Schwann cells to examine the effect of exogenous and endogenous MMP7. Meanwhile, the effects of MMP7 in nerve regeneration after sciatic nerve crush in vivo were observed. Furthermore, RNA sequencing and bioinformatic analysis of Schwann cells were conducted to show the molecular mechanism behind the phenomenon. In vitro studies showed that MMP7 significantly elevated the migration rate of Schwann cells but did not affect the proliferation rate of Schwann cells. In vivo studies demonstrated that increased level of MMP7 contributed to Schwann cell migration and myelin sheaths formation after peripheral nerve injury. MMP7-mediated genetic changes were revealed by sequencing and bioinformatic analysis. Taken together, our current study demonstrated the promoting effect of MMP7 on Schwann cell migration and peripheral nerve regeneration, benefited the understanding of cellular and molecular mechanisms underlying peripheral nerve injury, and thus might facilitate the treatment of peripheral nerve regeneration in clinic.

## Introduction

Peripheral nerve injury is a common clinical issue that substantially affects patients’ quality of life and leads to severe social and economic burdens [1]. Treatments of peripheral nerve injury, including nerve suturing, autologous nerve grafting, and tissue engineered nerve transplantation, facilitated the functional recovery of injured nerve [2, 3]. However, to date, the clinical effects of these therapies have not reached a satisfactory level [4, 5]. Gaining a deeper understanding of the cellular and molecular mechanisms underlying peripheral nerve injury will benefit the clinical treatment of peripheral nerve injury and thus is in a pressing need.

Emerging studies showed that Schwann cells, as the main glial cells in the peripheral nervous system, play significant roles during peripheral nerve regeneration. Following peripheral nerve injury, Schwann cells sense injury signal, switch to a proliferating state, migrate to the injured site to clear axon and myelin debris and build bands of Büngner. Schwann cells then re-differentiate to a myelinating state and ensheath regenerated axons [6, 7]. Meanwhile, Schwann cells also secret neurotrophic factors to propel axon regrowth as well as proteolytic enzymes to re-organize extracellular matrix and generate a suitable extrinsic environment for nerve regeneration [8–10].

Matrix metalloproteinases (MMPs) are secreted proteolytic enzymes that are capable of cleaving and degrading the extracellular matrix [11, 12]. MMPs are a family of ubiquitously expressed endopeptidases and can be functional classified to collagenases (MMP1, MMP8 and MMP13), gelatinases (MMP2 and MMP9), stromelysins (MMP3, MMP10 and MMP11), matrilysin (MMP7 and MMP26), metalloelastase (MMP12), enamelysin (MMP20), membrane-type MMPs (MMP14, MMP15, MMP16, MMP17, MMP24 and MMP25), and other MMPs (MMP19, MMP21, MMP23, MMP27 and MMP28) [13]. These members of the MMP family play critical roles in regulating cell behaviors, such as cell apoptosis, proliferation, migration and differentiation [14]. MMPs have also been linked to many pathological conditions including injuries to peripheral nervous system [15–17]. For example, it was demonstrated that gelatinases MMP2 and MMP9 were up-regulated after peripheral nerve injury and regulated the proliferation, migration, myelination and neurite-promoting potential of Schwann cells [10, 18–20].

Notably, our previously performed deep sequencing analysis showed that besides gelatinases MMP2 and MMP9, matrilysin MMP7 was significantly up-regulated in rat sciatic nerve segments after nerve injury as well [21]. However, the physiological roles of MMP7 remain largely unclear. Therefore, the aims of the current study were to determine the functional effects of MMP7 on the modulation of Schwann cell phenotype and the regeneration of injured peripheral nerves. For the first time, we reported that MMP7 contributed to the migration and myelination of Schwann cells during peripheral nerve regeneration.

## Materials and methods

### Primary Schwann cell isolation and culture

Primary Schwann cells were isolated from the sciatic nerve segments of neonatal Sprague-Dawley (SD) rats as previously described [22]. Briefly, rat sciatic nerve segments were surgically excised and treated with collagenase and trypsin. Collected cells were cultured in Dulbecco’s modified eagle medium (DMEM; Invitrogen) supplemented with 10% fetal bovine serum (FBS; Invitrogen), 1% penicillin and streptomycin (Invitrogen), 2 μM forskolin (Sigma), and 10 ng/ml heregulin β1 (HRG; Sigma) till confluence. Cultured cells were then treated with anti-Thy1.1 antibody (Sigma, St. Louis, MO, USA) and rabbit complement (Invitrogen, Carlsbad, CA, USA) to remove fibroblasts. Purified Schwann cells were grown in cell culture medium containing DMEM, 10% FBS, and 1% penicillin and streptomycin (Invitrogen) in a humidified 5% CO_2_ incubator at 37 °C. Cultured primary Schwann cells were passaged for no more than 2 passages prior to use.

### Schwann cell treatment

For MMP7 treatment, cultured Schwann cells were exposed to 10 nM recombinant active human MMP-7 matrilysin purified protein (CC1059; Merck Millipore, Billerica, MA). For cell transfection, cultured Schwann cells were transfected with siRNA against MMP7 (MMP7 siRNA1: GGGAAATGCAGAAGTTCTT, MMP7 siRNA2: GCAGGCATCCAGAAGTTAT, or MMP7 siRNA3: AAAGAGGAACAAGCTGTGA) or non-targeting negative control (Ribobio, Guangzhou, Guangdong, China) by using Lipofectamine RNAiMAX transfection reagent (Invitrogen) according to the manufacturer’s instructions. RNA was isolated from transfected cells, reverse transcribed to cDNA by using the Prime-Script reagent kit (TaKaRa, Dalian, Liaoning, China), and subjected to RT-PCR and Western blot experiments to verify transfection efficiency.

### Real-time RT-PCR

RT-PCR was conducted by using SYBR Green Premix Ex Taq (TaKaRa) on a StepOne Real-time PCR System (Applied Biosystems, Foster City, CA, USA). The relative expression level of MMP7 was calculated by using the 2^-ΔΔCt^ method with GAPDH as the internal control. The sequences of primer pairs were: MMP7 (forward) 5′-CAGGAAGCCGGAGAAGTGAC-3′ and (reverse) 5′-TCTCCGGCAAACCGAAGAAC-3′; and GAPDH (forward): 5′-CCTTCATTGACCTCAACTACATG-3′ and (reverse) 5′-CTTCTCCATGGTGGTGAAGAC-3′.

### Western blot

Protein extracts were prepared from cell cultures. Equivalent amounts of isolated protein were separated on 10% SDS-PAGE and transferred to PVDF membranes (Millipore, Bedford, MA, USA), The membranes were blocked with 5% nonfat dry milk in Tris-HCl buffered saline (TBS) at room temperature and probed with MMP7 (1:1000, Cell Signaling, Beverly, MA, USA) primary antibody, followed by horseradish peroxidase (HRP)-conjugated second antibody. Subsequently, these membranes was developed with enhanced chemiluminescence reagent (Cell Signaling, Beverly, MA, USA) and exposed to Kodak X-Omat Blue film (NEN life science, Boston, MA, USA). The immunoreactive bands were quantitatively analyzed using Grab-it 2.5 and Gelwork software.

### EdU proliferation assay

The proliferation rate of Schwann cells was examined by using EdU proliferation assay as previously described [22]. Briefly, Schwann cells were seeded on poly-L-lysine-coated 96-well plates at a density of 2 × 10^5^ cells/ml. 50 μM EdU was added to cell culture medium and Schwann cells were cultured for additional 24 h prior to fixation. Cell proliferation rate was determined by using a Cell-Light EdU DNA Cell Proliferation Kit (Ribobio) and was calculated as the ratio of the number of EdU-positive cells to the number of total cells. Images were obtained under a DMR fluorescence microscope (Leica Microsystems, Bensheim, Germany).

### Transwell migration assay

The migration ability of Schwann cells was examined by using Transwell migration assay as previously described [22]. 100 μl Schwann cells with 3 × 10^5^ cells/ml were resuspended in DMEM and added to the top chamber of a 6.5 mm Transwell with 8 μm pores (Costar, Cambridge, MA, USA). 500 μl cell culture medium was added to the bottom chamber which was pre-coated with 10 μg/ml fibronectin. At 12 or 24 h after cell culture, the upper surface of the top chamber was cleaned with a cotton swab while the bottom surface was stained with 0.1% crystal violet. Schwann cells adhering to the bottom surface were observed and counted under a DMR inverted microscope (Leica Microsystems).

### Animal surgery

Male, adult SD rats weighting 180-220 g were obtained from the Experimental Animal Center of Nantong University, Jiangsu, China. Animal experiments were conducted in accordance with the Institutional Animal Care Guidelines of Nantong University and ethically approved by the Administration Committee of Experimental Animals.

SD rats were anaesthetized intraperitoneally with a mixture of 85 mg/kg trichloroacetaldehyde monohydrate (RichJoint, Shanghai, China), 42 mg/kg magnesium sulfate (Xilong Scientific, Guangzhou, Guangdong, China), and 17 mg/kg sodium pentobarbital (Sigma). After anaesthetization, the sciatic nerve segments at 10 mm above the bifurcation into the tibial and common fibular nerves were crushed. 100 nM MMP7 recombinant protein was dissolved in 6 μl saline and injected perineurally at the injury site immediately after nerve injury. In the control group, 6 μl saline was injected perineurally at the injury site. Rats were sacrificed and sciatic nerve segments at the injury sites were collected at 1, 4, 7 and 14 days after surgery.

### Immunofluorescence staining

Cell cultures were prepared and fixed in 4% paraformaldehyde. Sciatic nerve segments were dissected, fixed in 4% paraformaldehyde, dehydrated in 30% sucrose at 4 °C, then cut and mounted onto microscope slides. Sciatic nerve sections and cell cultures were blocked with 5% goat serum for 30 min, incubated with primary antibodies overnight at 4 °C, and then incubated with fluorescent-dye-conjugated secondary antibodies for 1 h at room temperature. Primary antibodies included mouse anti-S100 antibody (1:1000 dilution, Sigma), rabbit anti-Ki67 antibody (1:200 dilution, Sigma), mouse anti-NF200 antibody (1:200 dilution, Sigma), rabbit anti-S100 antibody (1:200 dilution, Abcam), and Hoechst 33342 (1:5000 dilution, Life Technologies). Secondary antibodies included goat anti-mouse IgG-Alex-488 (1:500 dilution, Abcam), and goat anti-rabbit IgG-Cy3 (1:100 dilution, Abcam). Images were obtained under a fluorescence microscopy (Axio Imager M2, Carl Zeiss Microscopy GmbH, Jena, Germany).

### Transmission electron microscopy

SD rats were randomly separated into two groups and subjected to crush injury plus MMP7 recombinant protein injection, and crush injury plus saline injection. SD Rats were perfused with a fixative containing 1% paraformaldehyde and 1.25% glutaraldehyde at 14 days after surgery. Sciatic nerve segments were collected, fixed in 4% glutaraldehyde, and embedded in Epon 812 epoxy resin (Sigma). Ultrathin sections were obtained and stained with lead citrate and uranyl acetate. The morphology of sciatic nerve segments were observed under a transmission electron microscope (JEOL Ltd., Tokyo, Japan).

### RNA sequencing and bioinformatic analysis

Schwann cell treated with recombinant MMP7 were subjected to RNA isolation and sequencing analysis by using Illumina HiSeq™ 2500 (Gene Denovo Biotechnology Co., Guangzhou, China). High quality clean reads obtained by filtering raw reads containing adapters, more than 10% of unknown nucleotides, or more than 50% low quality bases were mapped to reference genome by TopHat2 (version 2.0.3.12) [23]. Gene abundances were quantified by RSEM [24] and normalized by Fragments Per Kilobase of transcript per Million mapped reads (FPKM) method. Differentially expressed genes with a fold change ≥2 and a false discovery rate (FDR) < 0.05 were screened and listed in Additional file 1: Table S1. Differentially expressed genes were analyzed by Database for Annotation, Visualization, and Integrated Discovery (DAVID) bioinformatic resources to identify enriched Gene ontology (GO) terms and Kyoto Enrichment of Genes and Genomes (KEGG) pathways. RNA-seq data used in this study have been deposited in the National Center for Biotechnology Information Sequence Red Archive (SRA) under the accession code SRP173072.

### Data analysis

Numerical results were presented as means ± SD. Student’s t-test was used for comparison between two groups and ANOVA was used for multiple comparisons. Statistical analysis was conducted by using Stata 7.0 software package (Stata Corp., College Station, TX, USA). A *p*-value< 0.05 was considered as statistically significant.

## Results

### MMP7 promoted the migration instead of the proliferation of Schwann cells

**Fig. 1:**
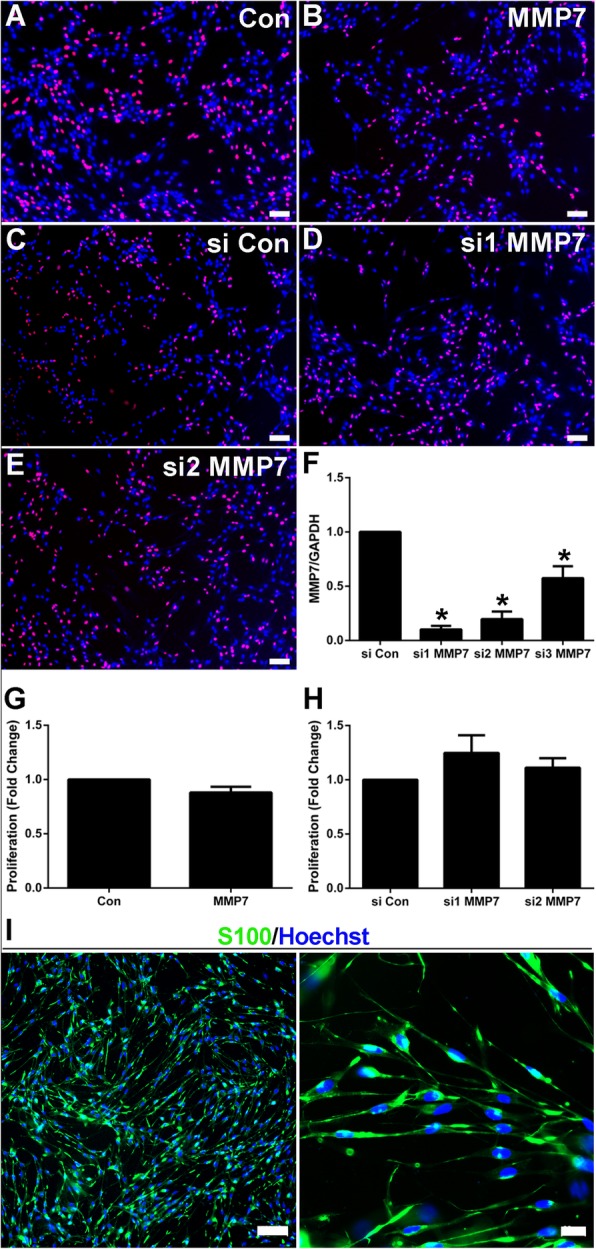
The in vitro effect of MMP7 on Schwann cell proliferation. **a**-**e** Representative cell proliferation images of (**a**) non-treated Schwann cells (Con), **b** Schwann cells treated with MMP7 recombinant protein (MMP7), **c** Schwann cells transfected with siRNA control (si Con), **d** Schwann cells transfected with MMP7 siRNA1 (si1 MMP7), and (**e**) Schwann cells transfected with MMP7 siRNA2 (si2 MMP7). Scale bars represented 100 μm. **f** Relative MMP7 mRNA expression after transfection with siRNA control, MMP7 siRNA1, MMP7 siRNA2, and MMP7 siRNA3. Asterisks indicated significant different from siRNA control, * *p*-value < 0.05, *n* = 3. **g** Summarized proliferation rates of non-treated Schwann cells and Schwann cells treated with MMP7 recombinant protein. *n* = 3. **h** Summarized proliferation rates of Schwann cells transfected with siRNA control, MMP7 siRNA1, and MMP7 siRNA2. *n* = 3. **i** Schwann cells’ identification by histochemical staining. Specific marker S100 of Schwann cells is green, and Hoechst of nucleus is blue. Scale bars represented 100 μm and 20 μm respectively

The functional effect of MMP7 on Schwann cell proliferation was first examined by EdU proliferation assay. Schwann cells exposed to MMP7 recombinant protein exhibited comparable cell proliferation rate as non-treated Schwann cells (Fig. 1a, b, and g), indicating that exogenous MMP7 did not significantly affect Schwann cell proliferation. Schwann cells were then transfected with siRNA against MMP7 to determine whether endogenous MMP7 level would affect Schwann cell proliferation. A total of three siRNAs (designated as MMP7 siRNA1, MMP7 siRNA2, and MMP7 siRNA3) were designed and used. Compared with the non-targeting negative control, transfected Schwann cells with all these three siRNAs robustly reduced MMP7 mRNA expression (Fig. 1f). MMP7 siRNA1 and MMP7 siRNA2, two siRNAs with higher silencing efficiencies, were subsequently used. Similarly, Schwann cells transfected with MMP7 siRNA1 or MMP7 siRNA2 did not exhibit altered proliferation rate as compared with cells transfected with the non-targeting negative control (Fig. 1c, d, e, and h).

**Fig. 2:**
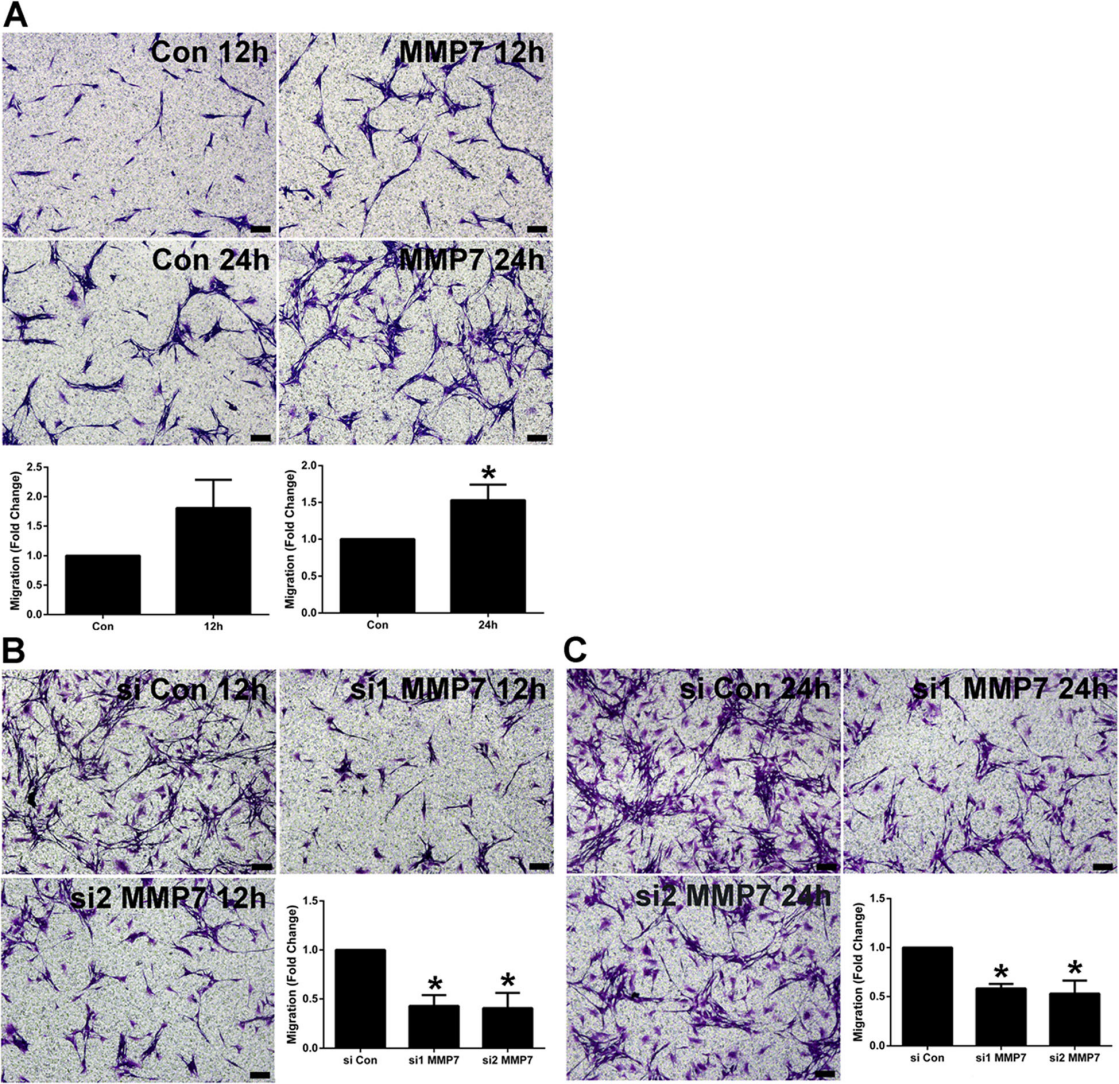
The in vitro effect of MMP7 on Schwann cell migration. **a** Representative cell migration images of non-treated Schwann cells (Con) and Schwann cells treated with MMP7 recombinant protein (MMP7) at 12 and 24 h after cell culture. Scale bars represented 100 μm. Histograms showed summarized migration rates of non-treated Schwann cells and Schwann cells treated with MMP7 recombinant protein. The asterisk indicated significant different from non-treated control, * *p*-value < 0.05, *n* = 3. **b** Representative cell migration images of Schwann cells transfected with siRNA control, MMP7 siRNA1, and MMP7 siRNA2 at 12 h after cell culture. Scale bars represented 100 μm. Histograms showed summarized migration rates of Schwann cells transfected with siRNA control, MMP7 siRNA1, and MMP7 siRNA2. Asterisks indicated significant different from siRNA control, * *p*-value < 0.05, *n* = 3. **c** Representative cell migration images of Schwann cells transfected with siRNA control, MMP7 siRNA1, and MMP7 siRNA2 at 24 h after cell culture. Scale bars represented 100 μm. Histograms showed summarized migration rates of Schwann cells transfected with siRNA control, MMP7 siRNA1, and MMP7 siRNA2. Asterisks indicated significant different from siRNA control, * *p*-value < 0.05, *n* = 3

Schwann cells exposed to endogenous MMP7 recombinant protein or transfected with siRNA against MMP7 were then seeded onto Transwell chambers to determine the effect of MMP7 on Schwann cell migration. There existed a relative larger number of migrated cells in Schwann cells treated with MMP7 recombinant protein at 12 h after culture (Fig. 2a). The amount of migrated cells in MMP7 treated Schwann cells was significantly larger as compared with non-treated cells at 24 h after culture, suggesting that MMP7 enhanced the migration ability of Schwann cells (Fig. 2a). In contrast, Schwann cells transfected with MMP7 siRNA1 and MMP7 siRNA2 showed suppressed migration ability at 12 and 24 h after culture (Fig. 2b and c). These in vitro outcomes demonstrated that MMP7 did not affect the proliferation of Schwann cells but promoted the migration of Schwann cells.

### MMP7 promoted the migration of Schwann cells from the proximal nerve segment after sciatic nerve injury

**Fig. 3:**
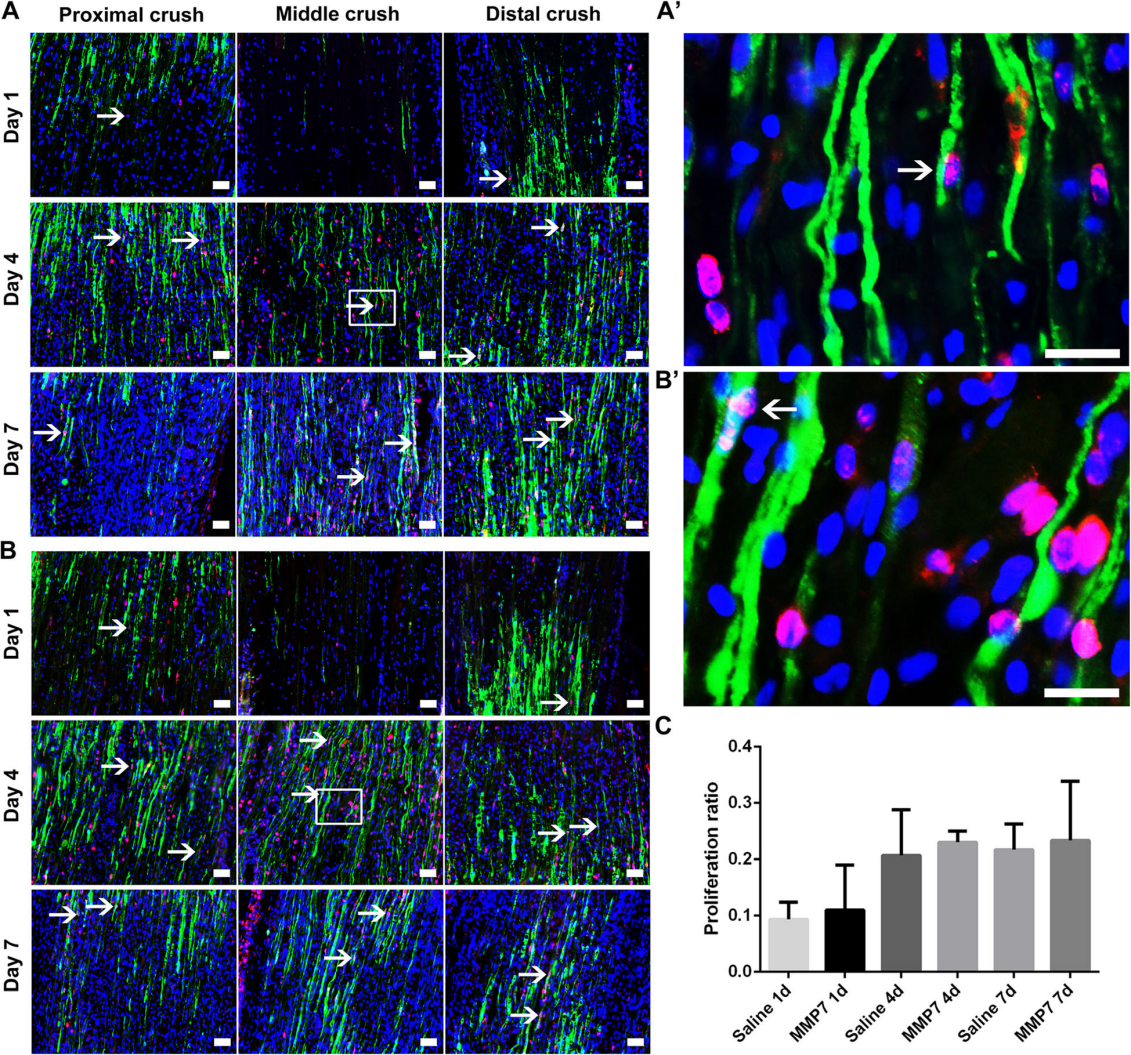
The in vivo effect of MMP7 on Schwann cell proliferation. **A** and **B** Representative immunohistochemistry images of the longitude sections of rat sciatic nerve segments treated with **A** saline control and **B** MMP7 recombinant protein. Rat sciatic nerve segments were collected at 1, 7 and 14 days after nerve crush. Green indicated S100 staining of Schwann cells, red indicated Ki67 staining of proliferating cells, and blue indicated Hoechst staining of cell nuclei. Arrows indicated proliferating Schwann cells. Scale bars represented 50 μm. Boxed areas in (**A** and **B**) were demonstrated in higher magnifications in (**A**’ and **B**′), respectively. Arrows indicated proliferating Schwann cells too. Scale bars represented 20 μm. **c** Histogram of proliferation ratio in rats injected with saline or MMP7 recombinant protein at 1, 7 and 14 days after nerve crush, *n* = 3

Rat sciatic nerve crush surgery was conducted and saline or MMP7 recombinant protein were directly injected into the injury site immediately after injury to determine the in vivo effect of MMP7 following sciatic nerve injury. Injured sciatic nerve segments were triple-immunostained with Schwann cell marker S100 (green), proliferation marker Ki67 (red) and cell nucleus marker Hoechst (blue), then proliferating Schwann cells were observed at the proximal site, distal site and middle crush site at 1, 4 and 7 days after nerve injury (Fig. 3). In the saline control group, a few proliferating Schwann cells could be detected at both the proximal site and the distal site at 1 day after nerve injury (Fig. 3a). More proliferating Schwann cells were observed at both sites at later time points (4 and 7 days after nerve injury) (Fig. 3a). And some proliferating Schwann cells were also detected at the middle crush site (Fig. 3A and A’). Similar as rats injected with saline, rats injected with MMP7 had a few proliferating Schwann cells at the injury site at 4 and 7 days (Fig. 3B and B’). Comparison results showed that there existed no significant difference in the number of proliferating Schwann cells in the saline group (Fig. 3A) and MMP7 treatment group (Fig. 3B) at all three tested time points (Fig. 3C), indicating that MMP7 did not influence Schwann cell proliferation after sciatic nerve injury. More other types of proliferating cells were also shown in the injury site in both two groups.

**Fig. 4:**
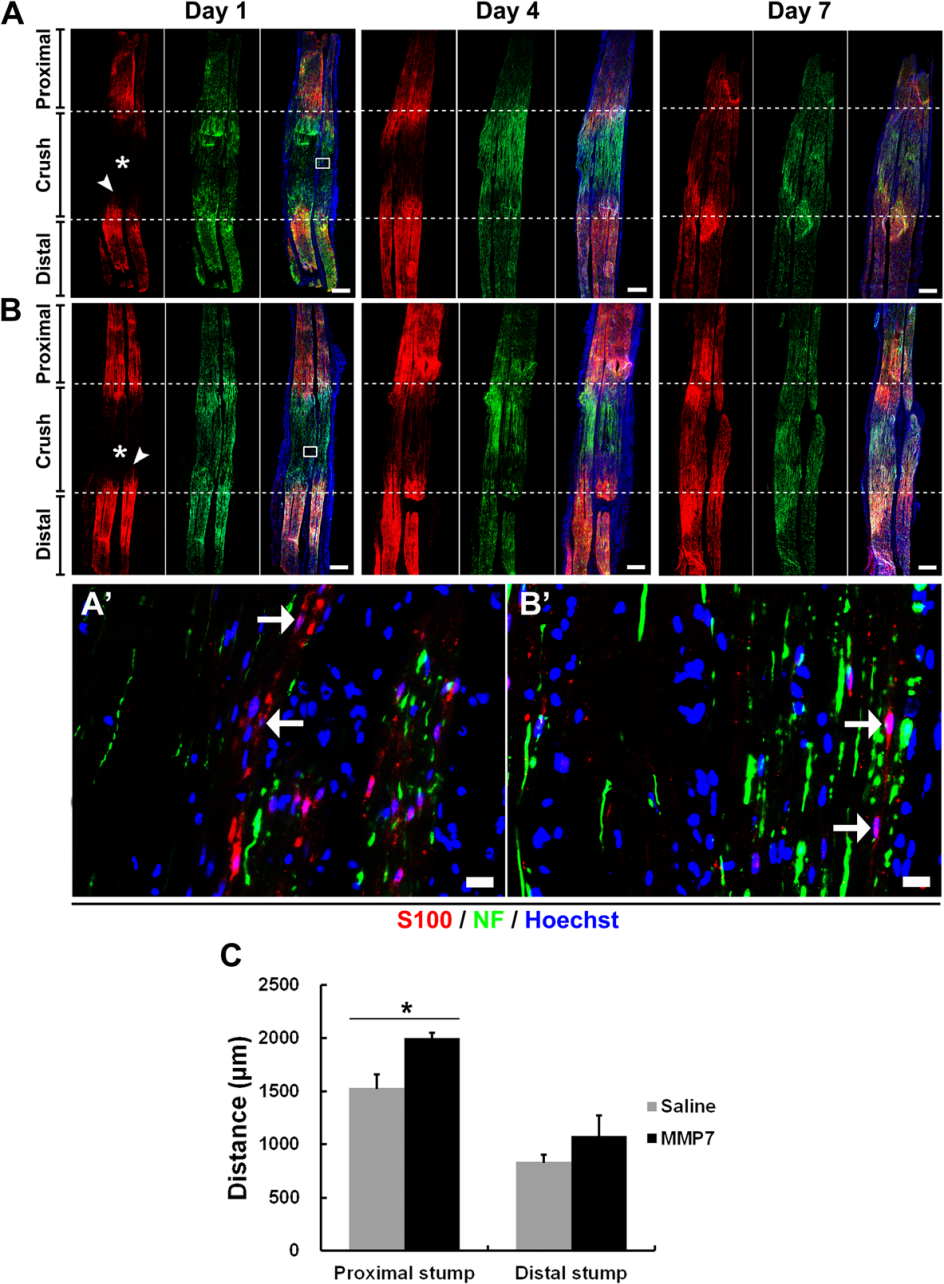
The in vivo effect of MMP7 on Schwann cell migration. **A** and **B** Representative immunohistochemistry images of the longitude sections of rat sciatic nerve segments treated with (**A**) saline control and (**B**) MMP7 recombinant protein. Rat sciatic nerve segments were collected at 1, 7 and 14 days after nerve crush. Red indicated S100 staining of Schwann cells, green indicated NF staining of axons, and blue indicated Hoechst staining of cell nuclei. Asterisks indicated migration from the proximal segment. Arrowheads indicated migration from the distal segment. Scale bars represented 500 μm. Boxed areas in (**A** and **B**) were shown in higher magnifications in (**A**’ and **B**’), respectively. Arrows indicated migrated Schwann cells. Scale bars represented 20 μm. **c** Summarized migration distances in rats injected with saline or MMP7 recombinant protein. Asterisk indicated significant different from saline control, * *p*-value < 0.05, *n* = 3

Injured sciatic nerve segments were then triple-immunostained with S100 (red), axon marker NF (green) and Hoechst (blue) to determine the migration of Schwann cells (Fig. 4). It was observed from the saline control group that at 1 day after nerve injury, some Schwann cells migrated toward the middle crush site from both the proximal site (marked in asterisk) and the distal site (marked in arrowhead). More Schwann cells migrated into the middle crush site at 4 days after nerve injury and the nerve gap was connected by migrated Schwann cells. And a clear band of Büngner was observed at 7 day after nerve injury (Fig. 4a). Compared with in the saline group, the migration ability of Schwann cells in rats treated with MMP7 recombinant protein was even higher, especially at 1 day after nerve injury (Fig. 4b). The distances of Schwann cell migration from both proximal and distal sites were measured and summarized. It was demonstrated that Schwann cells in MMP7-treated rats migrated a longer distance to the middle crush site, especially from the proximal site (Fig. 4c). NF staining suggested that axons at the distal sites were degenerated while axons at the proximal sites were regenerated after nerve injury. The lengths of regenerated axons were basically in consistent with the distances of migrated Schwann cells and in MMP7 treated rats, axons regenerated to a longer distance at 1 day after nerve injury (Fig. 4a and b).

### MMP7 promoted myelin sheath formation after sciatic nerve injury

**Fig. 5:**
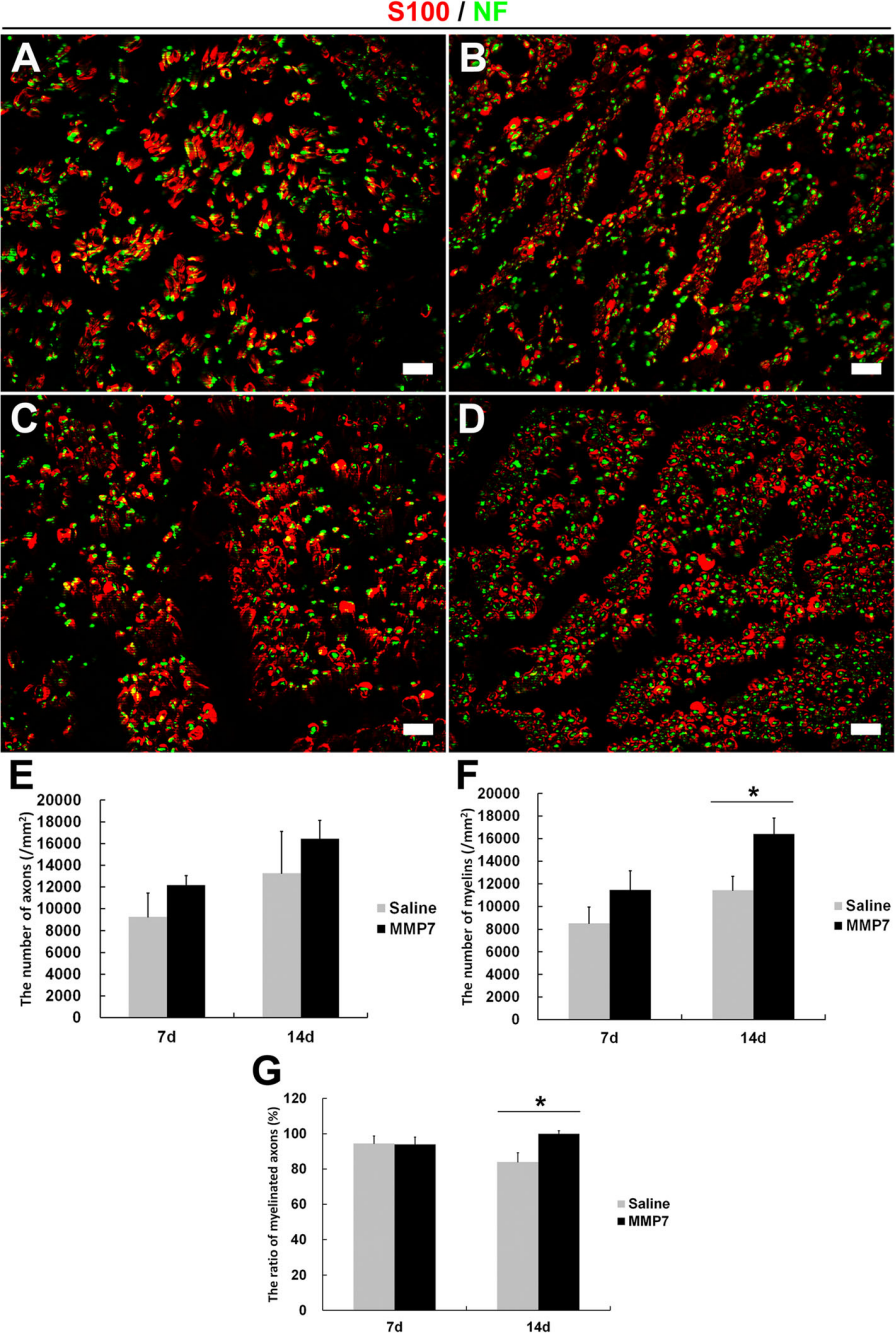
The in vivo effect of MMP7 on axon and myelin sheath regeneration. **a** and **b** Representative immunohistochemistry images of the cross sections of rat sciatic nerve segments (crush sites) treated with saline at (**a**) 7 days and (**b**) 14 days after nerve crush. Red indicated S100 staining of Schwann cells and green indicated NF staining of axons. Scale bars represented 20 μm. **c** and **d** Representative immunohistochemistry images of rat sciatic nerve segments (crush sites) treated with MMP7 recombinant protein at (**c**) 7 days and (**d**) 14 days after nerve crush. Red indicated S100 staining of Schwann cells and green indicated NF staining of axons. Scale bars represented 20 μm. **e** Summarized numbers of axons in rats injected with saline or MMP7 recombinant protein. **f** Summarized numbers of myelin sheaths in rats injected with saline or MMP7 recombinant protein. **g** Summarized ratio of myelinated axons in rats injected with saline or MMP7 recombinant protein. Asterisk indicated significant different from saline control, * *p*-value < 0.05, *n* = 3

Regenerated axons should be wrapped by migrated Schwann cells to achieve the functional recovery of injured nerves. In order to observe Schwann cell surrounding situation, besides observing the overall regeneration outcomes from the longitude aspect, cross sections of sciatic nerve segments were made at the crush sites and the morphological features of regenerated nerves were obtained. Immunohistochemistry staining with NF (green) and S100 (red) was performed at 7 and 14 days after nerve injury to evaluate axon regrowth and Schwann cell surrounding, respectively (Fig. 5). A higher density of axons and myelin sheaths was detected at 14 days (Fig. 5b and d) compared with at 7 days (Fig. 5a and c) in both saline group and MMP7-treated group. Quantitative comparison revealed that compared with the saline group (Fig. 5a and b), the number of axons was slightly, although not significantly, larger in the MMP7-treated group (Fig. 5c and d) at both time points (Fig. 5e). The number of myelin sheaths and also ratio of myelinated axons were considerably larger at 14 days in the MMP7 treated group than in the saline group (Fig. 5f and g).

**Fig. 6:**
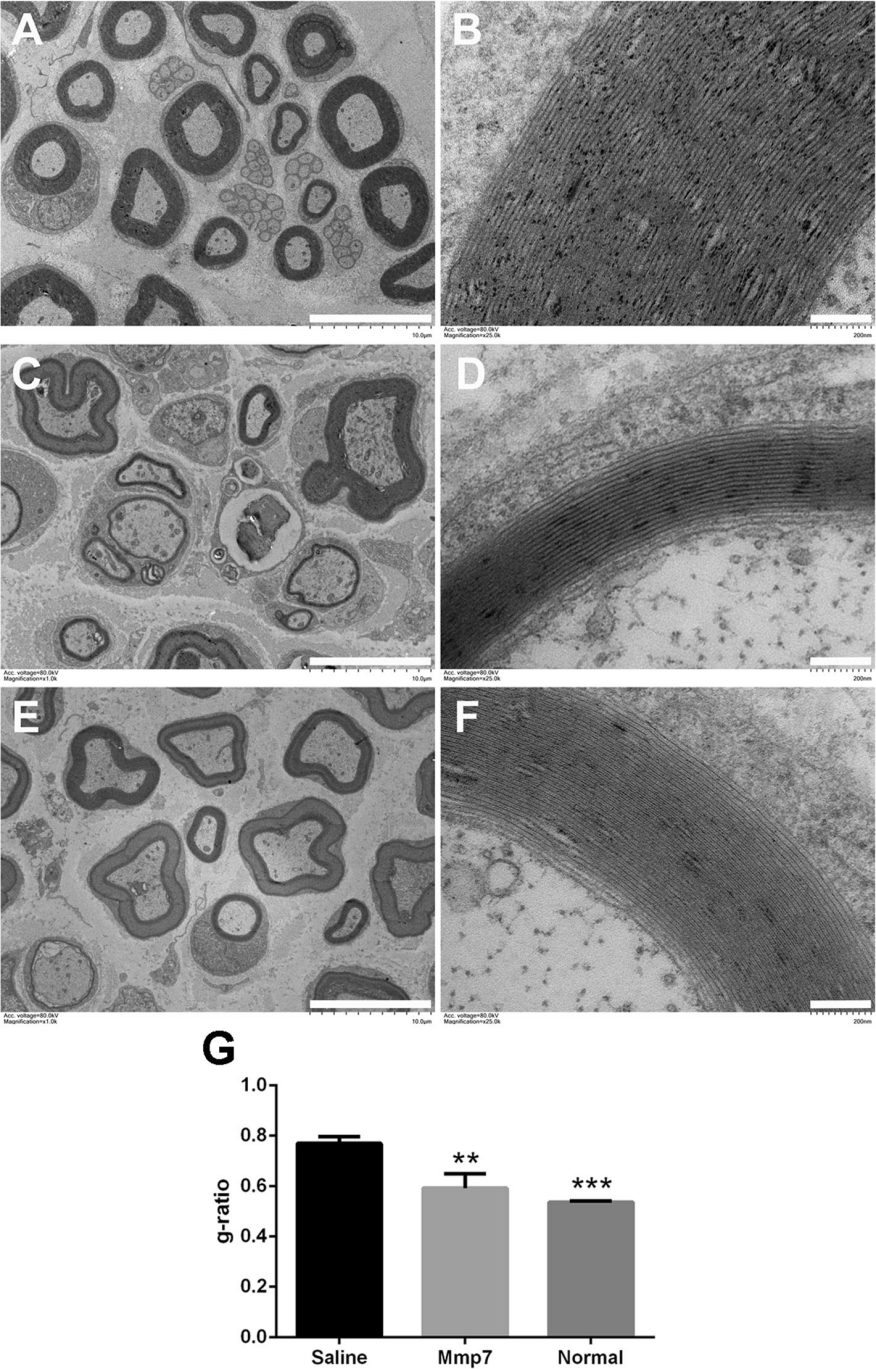
The in vivo effect of MMP7 on myelin sheath structure. **a** and **b** Representative transmission electron microscopy images of myelinated nerve fibers in the normal sciatic nerve. **c** and **d** Representative transmission electron microscopy images of myelinated nerve fibers in the sciatic nerve segments of rats treated with saline at 14 days after crush injury. **e** and **f** Representative transmission electron microscopy images of myelinated nerve fibers in the sciatic nerve segments of rats treated with MMP7 recombinant protein at 14 days after crush injury. Scale bars in (**a**, **c**, and **e**) represented 10 μm while scale bars in (**b**, **d**, and **f**) represented 200 nm. **g** Histogram of g-ratio (axon diameter to nerve fiber diameter) in rats injected with saline or MMP7 recombinant protein. Asterisk indicated significant different from saline control, ** *p*-value < 0.01, *** *p*-value < 0.001, *n* = 3

The formation of myelin sheaths was further observed under transmission electron micrography at 14 days after nerve injury. The normal sciatic nerve exhibited compact and dense myelin sheaths and clear basal membranes of Schwann cells (Fig. 6a and b). On the contrary, rats underwent sciatic nerve injury had relatively thinner myelin sheaths (Fig. 6c-e). Compared with rats injected with saline (Fig. 6c and d), rats injected with MMP7 recombinant protein showed larger number of myelin sheath layers and higher thickness of myelin sheaths (Fig. 6e, f and g), suggesting that MMP7 promoted the formation of myelin sheaths during peripheral nerve regeneration.

### MMP7 induced genetic changes in Schwann cells

**Fig. 7:**
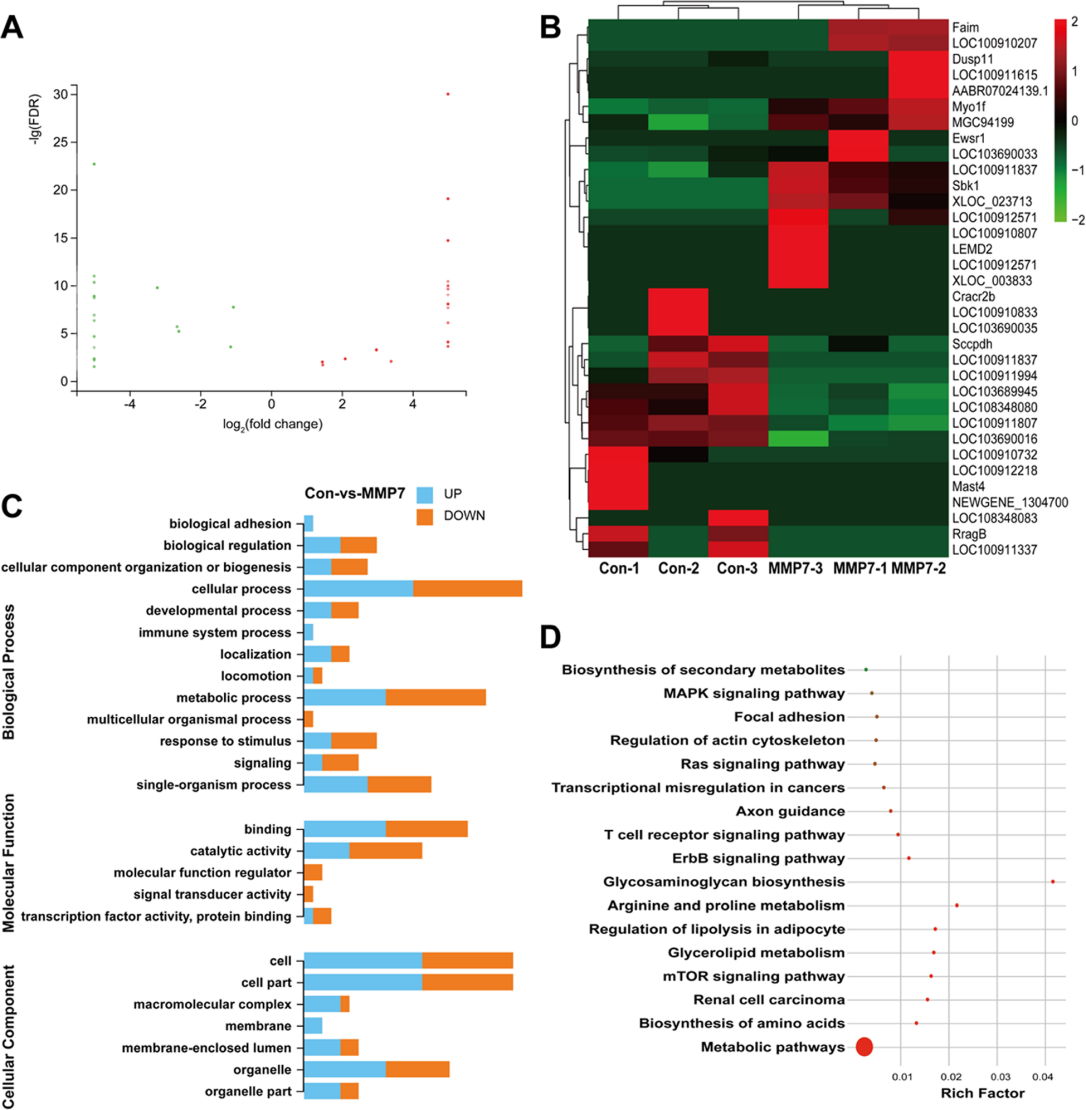
MMP7 mediated gene expression changes. **a** Scatter plot of differentially expressed genes in MMP7-treated Schwann cells. Red color indicated up-regulation while green color indicated down-regulation. **b** Heatmap of the expression levels of these differentially expressed genes. Red color indicated high expression level while green color indicated low expression level. **c** GO biological process, molecular function, and cellular component terms of differentially expressed genes. The length of bar graph was correlated with the numbers of differentially expressed genes. **d** KEGG pathways of differentially expressed genes. The size of the circle was correlated with the numbers of differentially expressed genes. The color of the circle was correlated with q-value (red color indicated low q-value)

To decipher molecular changes induced by MMP7, RNA samples were extracted from Schwann cells treated with or without MMP7 recombinant protein and subjected to RNA sequencing. A total of 34 genes were identified to be differentially expressed between the control group and MMP7-treated group with 17 genes up-regulated and 17 genes down-regulated (Fig. 7a and Additional file 1: Table S1). The expression levels of these differentially expressed genes were shown in a heatmap (Fig. 7b). GO analysis showed that in these differentially expressed genes, the most robust involved GO biological process terms were metabolic process (GO 0008152), biosynthesis process (cellular component organization or biogenesis, GO 0071840), and regulation of cell adhesion, including biological adhesion (GO 0022610), binding (GO 0005488), protein binding (GO 0005515), localization (GO 0051179), and locomotion (GO 0040011) (Fig. 7c). KEGG analysis demonstrated that significantly changing pathways were most involved in glycerolipid metabolism, biosynthesis of secondary metabolites, biosynthesis of amino acids, lycosaminoglycan biosynthesis, focal adhesion and actin cytoskeleton. Surprisingly, KEGG analysis results also showed that axon guidance pathway was activated (Fig. 7d).

## Discussion

MMPs have been highly associated with the development and regeneration of nervous system [25, 26]. Individual members of the MMP family may have detrimental and beneficial roles in nerve regeneration [27]. For example, MMP2 null mice exhibited impaired locomotion, rotarod performance, and grid walking after spinal cord injury, suggesting that gelatinase MMP2 had favorable effects on nerve regeneration [28]. Gelatinase MMP9 promoted Schwann cell migration [20], stimulated nerve growth factor-induced neurite elongation in regenerating peripheral nerve fibers [29], facilitated remyelination [30], and thus might have benefiting effects on nerve regeneration. On the other hand, inhibiting membrane-type MMP MMP14 or the application of broad-spectrum MMP inhibitor GM6001 would accelerate sensory axon regeneration after sciatic nerve crush, suggesting that MMP14 had adverse effects on nerve regeneration [27, 31].

In the current study, we examined the biological effects of matrilysin MMP7 by using primary cultured Schwann cells and rat sciatic nerve crush model. Outcomes from EdU proliferation assay showed that neither MMP7 recombinant protein nor MMP7 siRNA affect the proliferation rate of Schwann cells while outcomes from Transwell migration assay showed that MMP7 recombinant protein and MMP7 siRNA increased and decreased the migration ability of Schwann cells, respectively. Consistent with these in vitro observations, outcomes from in vivo studies showed that the direct application of MMP7 recombinant protein to the injured sciatic nerve site did not affect the proliferation of Schwann cells, but accelerated the migration of Schwann cells to the injured site, especially from the proximal site. Immunohistochemistry staining and transmission electron microscopy observations showed that treatment with MMP7 extensively increased the number of myelin sheaths as well as the thickness of myelin sheaths, suggesting that MMP7 also promoted Schwann cell myelination. The investigation of the biological effects of MMP7 on Schwann cell phenotype and peripheral nerve regeneration could be improved by further studies, including the examination of the in vitro and in vivo working efficiency of MMP7 recombinant protein and the determination of the electrophysiological and behavioral parameters. Overall, these observations offered the first biological investigation of MMP7 and highly supported the critical role of MMP7 in peripheral nerve regeneration.

Moreover, MMP7-induced molecular changes were also investigated by sequencing and bioinformatic analysis. At a threshold value of fold change ≥2 and a FDR < 0.05, only 34 genes, including 2 non-annotated genes (XLOC_023713 and XLOC_003833) were found to be differentially expressed after MMP7 exposure. GO terms (biological process, molecular function, and cellular component) and KEGG pathways of these differentially expressed genes were analyzed to screen critical biological activities. GO cellular component analyses showed that these differentially expressed genes were mainly related with cell (GO 0005623), cell part (GO 0044464), organelle (GO 0043226), organelle part (GO 0044422) and membrane (GO 0016020). Extracellular matrix-related GO cellular component terms such as extracellular space (GO 0005615), extracellular region (GO 0005576), extracellular region part (GO 00044421), and extracellular matrix (GO 0031012), were not identified here although MMPs were known to be highly related with the tissue remodeling and the dynamic regulation of the extracellular matrix [32, 33]. A possible reason might be that an in vitro cell culture model was used to perform sequencing and the majority of gene changes reflected intracellular changes of Schwann cells. Additional experiments such as ELISA measurements of the extracellular components could be conducted in the future to determine extracellular changes after MMP7 treatment. GO biological process analyses showed that many differentially expressed genes were involved in metabolic and biosynthesis process, and also regulation of cell adhesion. Consistently, KEGG pathway analysis showed that numerous metabolism, biosynthesis, adhesion and cytoskeleton-related pathways were enriched. These analysis outcomes indicated the significant involvement of metabolic process and biosynthesis process after MMP7 exposure. Meanwhile, outcomes from GO and KEGG analysis notably revealed that some internal movement and migration-related biological activities of cells such as localization, locomotion, focal adhesion and actin cytoskeleton, were also highly enriched. KEGG pathway axon guidance was also involved. The biological changes above may be related to MAPK and Ras signaling pathways. Changes of genes in these biological activities from both extracellular and intracellular aspects might affect cellular movement and migration, induce MMP7-mediated Schwann cell migration, and promote axon regrowth and muscle reinnervation.

Collectively, our current study fully demonstrated the promoting effect of MMP7 on Schwann cell migration and myelin sheath formation and preliminary revealed the underlying genetic changes. These outcomes might help to deepen our understanding of the molecular mechanisms underlying peripheral nerve repair and regeneration and contribute to the identification of novel clinical targets of the treatment of peripheral nerve injury.

## Conclusions

our current study revealed the promoting effect of MMP7 on Schwann cell migration and peripheral nerve regeneration, benefited the understanding of cellular and molecular mechanisms underlying peripheral nerve injury, and thus might facilitated the treatment of peripheral nerve injury.

## Supplementary information

**Additional file 1: Table S1.** List of differentially expressed genes in MMP7-treated Schwann cells. Gene ID, symbol, reads, *p*-value, FDR, gene description, and involved GO terms and KEGG pathways of differentially expressed genes were listed.

## Data Availability

Sequencing data were uploaded to NCBI database (accession number SRP173072). Data that support the findings of this study are available from the corresponding author upon reasonable request.

## Abbreviations

DMEM: Dulbecco’s modified eagle medium
FBS: Fetal bovine serum
FDR: False discovery rate
FPKM: Fragments Per Kilobase of transcript per Million mapped reads
GO: Gene ontology
KEGG: Kyoto Enrichment of Genes and Genomes
MMP: Matrix metalloproteinase
MMP7: Matrix metalloproteinase 7
SD: Sprague-Dawley
